# The Therapeutic Potential of Targeting Cytokine Alarmins to Treat Allergic Airway Inflammation

**DOI:** 10.3389/fphys.2016.00214

**Published:** 2016-06-14

**Authors:** Chandler B. Sy, Mark C. Siracusa

**Affiliations:** Department of Medicine, Center for Immunity and Inflammation, New Jersey Medical School, Rutgers-The State University of New JerseyNewark, NJ, USA

**Keywords:** allergic airway inflammation, asthma, thymic stromal lymphopoietin, biologics, alarmins

## Abstract

Asthma is a heterogeneous disorder that results in recurrent attacks of breathlessness, coughing, and wheezing that affects millions of people worldwide. Although the precise causes of asthma are unclear, studies suggest that a combination of genetic predisposition and environmental exposure to various allergens and pathogens contribute to its development. Currently, the most common treatment to control asthma is a dual combination of β2-adrenergic receptor agonists and corticosteroids. However, studies have shown that some patients do not respond well to these medications, while others experience significant side effects. It is reported that the majority of asthmas are associated with T helper type 2 (T_H_2) responses. In these patients, allergen challenge initiates the influx of T_H_2 cells in the airways leading to an increased production of T_H_2-associated cytokines and the promotion of allergy-induced asthma. Therefore, biologics that target this pathway may provide an alternative method to treat the allergic airway inflammation associated with asthma. As of now, only two biologics (omalizumab and mepolizumab), which target immunoglobulin E and interleukin-5, respectively, are FDA-approved and being prescribed to asthmatics. However, recent studies have reported that targeting other components of the T_H_2 response also show great promise. In this review, we will briefly describe the immunologic mechanisms underlying allergic asthma. Furthermore, we will discuss the current therapeutic strategies used to treat asthma including their limitations. Finally, we will highlight the benefits of using biologics to treat asthma-associated allergic airway inflammation with an emphasis on the potential of targeting cytokine alarmins, especially thymic stromal lymphopoietin.

## Introduction

Asthma is a heterogeneous disorder characterized by airflow obstruction, bronchial hyper-responsiveness, and airway inflammation, resulting in recurrent attacks of breathlessness, coughing, and wheezing (Busse and Lemanske, 2001). According to the World Health Organization, an estimated 235 million people currently suffer from asthma (Asthma, 2015a). The prevalence of asthma has dramatically increased in developed countries over the past decade (Asthma, 2015b). In the United States alone approximately 9.3% of all children and 8% of all adults currently have asthma, for a total of 25 million people. Additionally, asthma exerts a tremendous economic burden, costing the US tens of billions of dollars in medical costs, lost school and work days, and early deaths (Asthma, 2015b). Thus, the development of effective therapies to treat asthma is of utmost importance.

Although the precise causes of asthma are unclear, studies suggest that a combination of genetic predisposition and environmental exposure to various allergens and pathogens contribute to its development (Gilmour et al., 2006; Brauer et al., 2007; Vercelli, 2008; Bush and Peden, 2009). Risk factors thought to be associated with asthma include exposure to inhaled substances such as both indoor (house dust mite) and outdoor allergens (pollen), tobacco smoke, chemical irritants, and certain types of air pollution (Gilmour et al., 2006; Brauer et al., 2007; Bush and Peden, 2009). In addition, exposure to certain respiratory viruses early in life may contribute to the development of asthma at a later age. For instance, infants and young children who suffer from wheezing illnesses caused by rhinovirus infections were found to be more likely to develop asthma by age 6 (Jackson et al., 2008). Similarly, severe lower respiratory viral infections during infancy were found to promote asthma in children at high atopic risk (Kusel et al., 2007). Finally, specific polymorphic variants of several genes including a disintegrin and metalloproteinase (ADAM) 33 (Holgate et al., 2007), G protein–coupled receptor for asthma susceptibility (GPRA; Laitinen et al., 2004), and human leukocyte antigen (HLA)-G (Nicolae et al., 2005) have been shown to be associated with asthma susceptibility. Collectively, these studies suggest that asthma is a remarkably complex disease which can be promoted by several distinct factors. The complexity of asthma and the obvious importance of both genetic and environmental factors in its development suggest that diverse treatment strategies may be necessary in order to target the pathways that promote specific forms of the disease.

Currently, the most common treatment to control asthma is a combination of β2-adrenergic receptor agonists, which relax airway smooth muscle, and corticosteroids, which reduce inflammation of the airways (National Heart Lung and Blood Institute, 2007). Anti-leukotrienes are also often added to the treatment regimen when β2-agonists and corticosteroids fail to adequately control the symptoms. Although this therapeutic program is generally effective for most patients, there are several concerns. Studies have shown that some patients with severe asthma do not respond as well to β2-adrenergic receptor agonists or corticosteroids (Chan et al., 1998), In some cases, this can be attributed to single nucleotide polymorphisms in specific asthma-related genes (Drazen et al., 1999; Israel et al., 2000; Sampson et al., 2000; Tantisira et al., 2004). Moreover, most likely because of their unspecific mechanisms of actions, inhaled corticosteroids may cause serious side effects, especially if taken systemically or in large doses (Chung and O'Byrne, 2003; Dahl, 2006). Therefore, the development of biologics that provide a more targeted treatment option would greatly benefit patients who fail to respond to traditional therapeutics or are experiencing significant side effects. The purpose of this article is to describe the potential benefits of employing biologic therapies and to highlight the cellular pathways they may target. First, we will review the immunologic mechanisms known to promote airway inflammation in the context of allergic asthma. Next, we will describe conventional treatment options and review their benefits, limitations, and possible side effects. Finally, we will introduce the potential of biologic therapies in treating allergic airway inflammation and highlight the cellular mechanisms through which they may operate.

## Immunologic mechanisms underlying asthma

**Figure 1:**
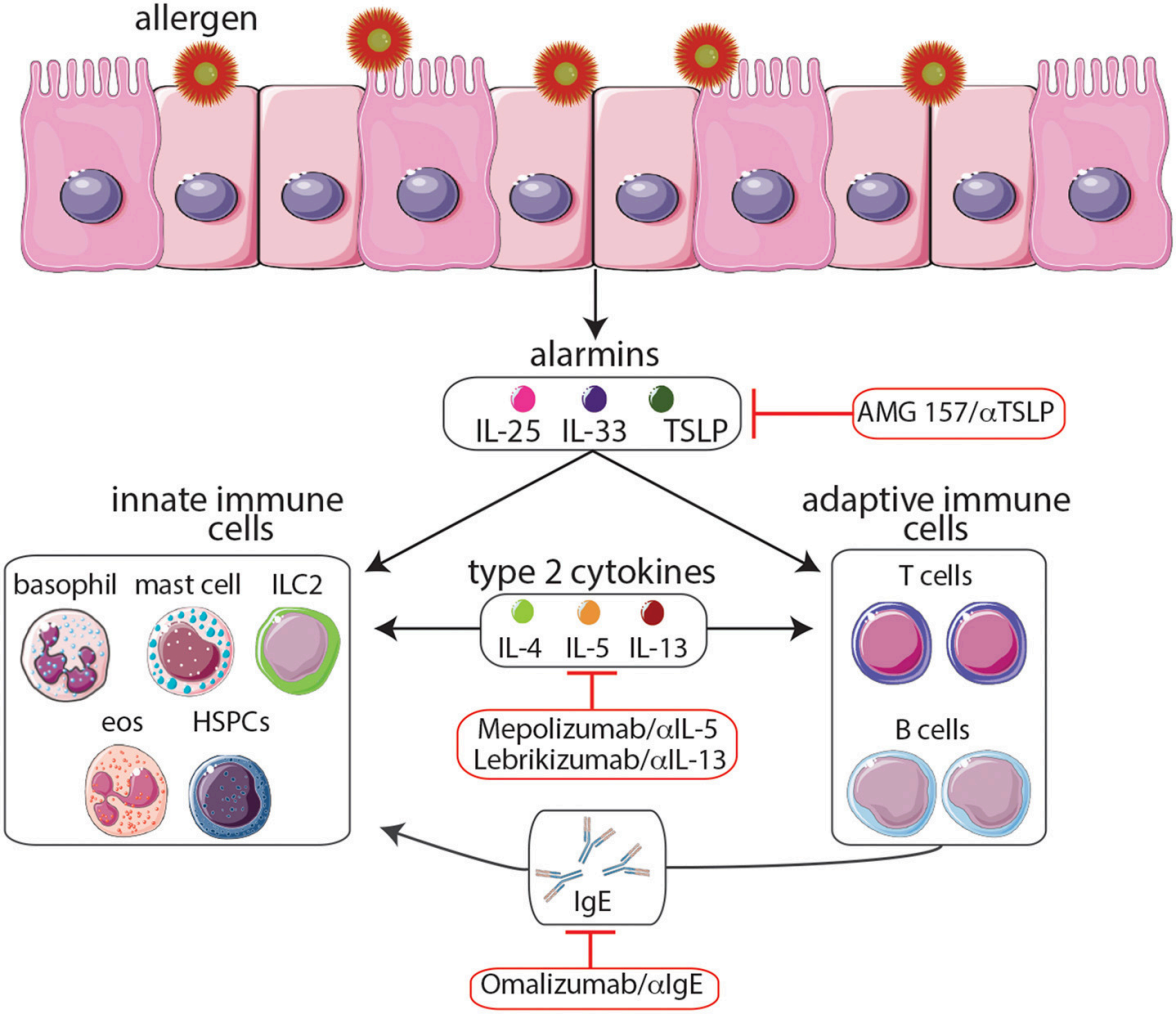
**Following exposure to allergens, epithelial cells release cytokine alarmins including thymic stromal lymphopoietin (TSLP), IL-25, and IL-33**. Cytokine alarmins promote the production of type 2 cytokines (IL-4, IL-5, and IL-13) by immune cells of both the innate and adaptive immune systems. Moreover, crosstalk between these two branches further promotes T_H_2 cytokine-mediated inflammation including production of IgE and the expansion of eosinophil populations, events that contribute to asthma pathogenesis. The anti-IgE monoclonal antibody omalizumab and the anti-IL-5 monoclonal antibody mepolizumab, the only biologics which have been approved by the FDA, provide effective long-term control and reduce the steroid requirement in patients with severe allergic asthma. Moreover, biologics targeting TSLP and IL-13 are currently undergoing clinical trials. Although biologics targeting the cytokine alarmins IL-25 and IL-33 have shown promise in murine studies, further studies are needed to determine their therapeutic potential. The development of biologics to treat allergy-associated asthma will aid in the eventual goal of creating novel personalized treatment regimens.

There are several phenotypes of asthma, each with its own natural history, clinical, and physiological features, pathobiology, and response to different therapies (Wenzel, 2012). The complex immunologic mechanisms responsible for promoting these various forms of asthma have been extensively discussed (Cohn et al., 2004). However, for the purpose of this article, we will briefly summarize the mechanisms underlying asthma, focusing on the role of T helper type 2 (T_H_2)-associated cytokines in the promotion of asthma, and highlight the therapeutic potential of targeting these pathways (Figure 1). The majority of asthmas are associated with T helper type 2 responses (Wenzel, 2012). In asthmatic patients, allergen challenge initiates the influx of T_H_2 cells in the airways leading to an increased production of T_H_2-associated cytokines including IL-4, IL-5, and IL-13 which promote the detrimental inflammation associated with asthma (Lloyd and Hessel, 2010). IL-4 production causes B cells to undergo IgE isotype switching resulting in an increase in circulating IgE, which sensitizes mast cells, eosinophils, and basophils to allergen exposure. Upon secondary exposure to the allergen, degranulation of these cells results in the release of histamine and reactive oxygen species leading to smooth muscle contraction, mucous secretion, and vasodilatation (Hamid and Tulic, 2009). In addition, IL-5 promotes the growth, maturation, and activation of eosinophils, which release potent cytotoxic proteins responsible for tissue destruction in the airways (Leckie et al., 2000). Finally, IL-13 also enhances IgE isotype switching, increases epithelial permeability, stimulates mucus production, and promotes airway hyper-responsiveness (Ingram and Kraft, 2012). Collectively, the activation of T_H_2 cells and the production of T_H_2-associated cytokines represent critical effector functions that are capable of promoting an asthmatic response.

The known role of T_H_2 cells and type 2 cytokines in promoting inflammation and asthma pathogenesis make the pathways that regulate these responses prime targets for anti-asthma therapies. Recent studies have found that cytokine alarmins, including thymic stromal lymphopoietin (TSLP), IL-25, and IL-33, produced by epithelial cells and some hematopoietic cells play important roles in the promotion of T_H_2 cell development and the initiation of asthma pathogenesis (Paul and Zhu, 2010). Therefore, therapies that target these molecules may hold the most promise as they could potentially shut down multiple components of the T_H_2 response responsible for asthma. In summary, as our knowledge of T_H_2 pathways increases, we are identifying new molecular targets which are involved in the initial stages of asthma pathogenesis. We will discuss the therapeutic potential of targeting these molecules in subsequent sections.

## Current therapeutic strategies

The current pharmacological agents used to treat asthma are focused on relieving airway obstruction and reducing chronic airway inflammation (National Heart Lung and Blood Institute, 2007). Inhaled β2-adrenergic receptor agonists are the most commonly used bronchodilators employed to treat asthma because of their rapid and potent effects in relaxing airway smooth muscle via direct stimulation of β2-receptors on airway smooth muscles (Cazzola et al., 2011). The most commonly used anti-inflammatory agents used to treat asthma consist of inhaled corticosteroids, which are beneficial but can have significant side effects. In addition, anti-leukotrienes, which have both anti-inflammatory and bronchoprotective effects, are used to alleviate symptoms either by themselves or in combination with inhaled glucocorticosteroids by inhibiting essential enzymes required for leukotriene production or by blocking their effects on target cells such as bronchial smooth muscle (Dahlén, 2006; National Heart Lung and Blood Institute, 2007). Despite their overall effectiveness, many current treatments focus heavily on targeting late phase events that promote inflammation, including histamine production and airway hyper-responsiveness, rather than on the initiation of the T_H_2 response (Holgate et al., 2007). Understanding the innate immune mechanisms that initiate T_H_2 cytokine-mediated inflammation would allow for the development of novel and more effective drugs that target the early stages of asthma pathogenesis.

Despite the efficacy of current therapies for most patients with asthma, clinical studies have shown that there is a variable response. In around 5–10% of people with asthma, the disease symptoms remain inadequately controlled even after treatment with inhaled corticosteroids, β2-adrenergic receptor agonists, and oral leukotriene inhibitors (Pelaia et al., 2012). Moreover, a retrospective study found that steroid insensitive (SI) asthma is quite common (25%) in adolescents with severe asthma (Chan et al., 1998). One possible reason for this variation in responses to asthma treatments are polymorphic variants in specific asthma-related genes; in fact previous pharmacogenetic studies suggest that genetics may contribute to as much as 60–80% of the variability observed in treatment response (Weiss et al., 2006). For example, it has been reported that variation in the corticotropin-releasing hormone receptor 1 (*CRHR1*) was associated with an enhanced response to corticosteroid therapy (Tantisira et al., 2004). Similarly, a single nucleotide polymorphism in the LTC4 synthase gene promoter may be associated with asthma that responds well to treatment with leukotriene receptor antagonists (Sampson et al., 2000). However, there are also polymorphic variants which are associated with a decreased response to certain pharmacological agents. Patients who were found to be homozygous for arginine at codon 16 of the β2-adrenergic receptor did not respond as well to the β2-adrenergic receptor agonist albuterol compared to patients without this polymorphism (Israel et al., 2000). Furthermore, sequence variants of the core promoter of *ALOX5*, the gene that codes for 5-lipoxygenase, an essential enzyme for leukotriene production, were associated with decreased clinical responses to ALOX5 inhibitors (Drazen et al., 1999). Collectively, these studies highlight the need for novel treatment options for patients who are found to be non-responsive to current therapies.

In addition to limitations of efficacy, the side effects of the most common therapeutics currently used to treat and control asthma represent another major concern. For example, long-term use of inhaled corticosteroids has been associated with several negative consequences including growth suppression, reduced bone density, glaucoma, cataracts, and skin thinning (Dahl, 2006). However, the severity and permanence of these side effects is still up for debate. For instance, although it is known that asthmatic children treated with inhaled corticosteroids experience suppressed growth before puberty, whether final adult height is affected is still unclear (Doull, 2004; Kelly et al., 2012). Similarly, whether patients who take inhaled corticosteroids suffer from a permanent reduction in bone density and are at an increased risk for osteopenia has not been determined (Peters, 2006; Kelly et al., 2008). Interestingly, several studies have suggested that inhaled corticosteroids, even when taken at relatively low doses, increase the risk of developing posterior subcapsular cataracts (Ernst et al., 2006; Wang et al., 2009). Additionally, although side effects are not common with inhaled β2-adrenergic receptor agonist therapy, it is reported that muscle tremors, tachycardia, and palpitations can occur in patients undergoing treatment (Chung and O'Byrne, 2003). In summary, although the long-term effects of current treatments for asthma remain up for debate and are most likely dependent on the dosage and duration of the treatment, new therapies would greatly benefit patients suffering from asthma, especially those who would otherwise require a high dosage of inhaled corticosteroid and β2-adrenergic receptor agonists.

## The benefits of biologics

While many of the therapies highlighted above target non-specific inflammatory pathways, biologics may offer physicians a personalized and more specific approach to treating allergic airway inflammation. To date, the only two biologics that are FDA-approved and being used to treat asthma are the humanized monoclonal IgE-targeting antibody omalizumab (Xolair) and the humanized monoclonal IL-5-targeting antibody mepolizumab (Nucala; Figure 1). Omalizumab works by binding to IgE, preventing its interaction with the high-affinity FcεRI expressed by mast cells, basophils, eosinophils, and dendritic cells, all of which are important in the pathogenesis of asthma (Busse and Lemanske, 2001). Studies have found that omalizumab provides effective long-term control and reduces the steroid requirement in patients with severe allergic asthma; additionally, omalizumab was found to be well tolerated (Soler et al., 2001; Lanier et al., 2003). Mepolizumab, works by targeting IL-5, an important T_H_2 cytokine that promotes the growth, maturation, and activation of eosinophils (Leckie et al., 2000). Mepolizumab was found to significantly reduce asthma exacerbations as measured by a variety of parameters including forced expiratory volume in 1 second (FEV1) and scores on the St. George's Respiratory Questionnaire and the 5-item Asthma Control Questionnaire (ACQ5; Leckie et al., 2000; Ortega et al., 2014). In addition, the safety profile of mepolizumab was similar to that of the placebo (Ortega et al., 2014).

In addition to anti-IgE and anti-IL-5 therapies, antibodies against the T_H_2-associated cytokine IL-13 may also hold great therapeutic potential. IL-13 produced in the airway promotes the survival and migration of eosinophils, the activation of macrophages, the production of mucus by airway epithelial cells, the transformation of airway fibroblasts to myofibroblasts leading to collagen deposition and tissue remodeling, and airway hyper-responsiveness, all of which are key features of allergic asthma (Ingram and Kraft, 2012). It is reported that lebrikizumab, a humanized monoclonal anti-IL-13 antibody that specifically binds to IL-13 and inhibits its function, was associated with improved lung function as measured by FEV1 but did not reduce asthma exacerbations or symptoms as measured by the ACQ5 (Corren et al., 2011). Additionally, the study found that periostin, a matricellular protein produced by bronchial epithelial cells in response to IL-13, could be used to predict patient response to lebrikizumab (Corren et al., 2011). This finding is especially important as different biologics would be expected to be more effective in treating different asthma phenotypes (Wenzel, 2012). Although treatments that inhibit the activity of T_H_2-associated cytokines and IgE show great potential, they target the later stages of the T_H_2 response, after T_H_2 and other inflammatory cells have already been recruited to and are active in the airways. Therefore, it is possible that targeting the more upstream events of asthma pathogenesis may represent a more effective strategy.

For this reason, thymic stromal lymphopoietin (TSLP) is an especially promising target. TSLP is a tissue-derived cytokine which is released by bronchial epithelial cells; consequently its effects are upstream of T_H_2-associated cytokine production by T_H_2 cells, mast cells, and basophils (Figure 1). TSLP has been found to be overexpressed in patients with asthma, atopic dermatitis, and food allergies, and specific polymorphic variants have been found to be associated with asthma and airway hyper-responsiveness (Ying et al., 2005; He et al., 2009). In addition, TSLP has been found to be crucial for driving T_H_2 cytokine-mediated inflammation in multiple murine model systems (Siracusa et al., 2011; Pelaia et al., 2012). For example, TSLP knockout mice fail to develop an inflammatory lung response to inhaled antigens in a murine model of allergic asthma (Al-Shami et al., 2005). Furthermore, it has been demonstrated that dendritic cells that are activated by TSLP prime naïve CD4^+^ T cells to differentiate into T_H_2 cells and produce the T_H_2 cell-attracting cytokines TARC (thymus and activation-regulated chemokines) and MDC (macrophage-derived chemokines; Soumelis et al., 2002; Ito et al., 2005). Even in the absence of T_H_2 cells, TSLP can act synergistically with IL-1 and tumor necrosis factor and stimulate mast cells to produce high levels of T_H_2 cytokines in response to inflammatory cytokines or physical injury (Allakhverdi et al., 2009). Moreover, TSLP has been shown to enhance T_H_2-associated cytokine expression in human innate helper 2 cells (ILC2) from peripheral blood and nasal polyps and to activate mouse lung and skin ILC2s (Halim et al., 2012; Mjösberg et al., 2012; Kim et al., 2013). TSLP also uniquely promotes T_H_2 responses by acting on progenitor cells. For example, circulating human CD34+ progenitor cells were found to express the TSLP receptor and in response to this cytokine, rapidly release high levels of proinflammatory T_H_2-associated cytokines and chemokines (Allakhverdi et al., 2009). Additionally, TSLP also appears to influence the hematopoietic programming of progenitor cells in the bone marrow. For example, it is reported that TSLP acts on bone marrow-resident progenitors to elicit the expansion of a functionally distinct population of basophils that promote T_H_2 cytokine-mediated inflammation (Siracusa et al., 2011). Therefore, the ability of TSLP to promote inflammation via targeting both innate and adaptive immune cells as well as altering hematopoiesis suggests it may represent a potent therapeutic target. In fact, a recent study found that treating patients with mild allergic asthma with AMG 157, a human anti-TSLP monoclonal antibody, reduced allergen-induced bronchoconstriction and airway inflammation both before and after allergen challenge; moreover there was no significant difference in the number of adverse events between the AMG 157 and the placebo group (Gauvreau et al., 2014). Although more studies need to be done to assess the clinical benefits of anti-TSLP antibody treatment, these initial results indicate that targeting TSLP may be an effective approach for treating allergic airway inflammation. Although the specific immunologic pathways involved in the initiation of asthma pathogenesis remain to be fully defined, gaining a better understanding of these pathways will guide the development of novel therapeutics that target multiple components of the T_H_2 response responsible for allergic asthma.

## Targeting additional cytokine alarmins

As mentioned previously, the cytokine alarmins IL-25 and IL-33 are similar to TSLP in that they also play important roles in the promotion of asthma pathogenesis and act upstream of multiple components of the T_H_2 response (Figure 1; Paul and Zhu, 2010; Beale et al., 2014). One study, which employed a murine model of T_H_2-type/eosinophilic allergic asthma, reported that IL-25 induces local inflammation by activating airway structural cells including epithelial cells as well as immune cells such as T_H_2 cells and eosinophils that drive airway inflammation (Suzukawa et al., 2012). Moreover, IL-25 deficiency resulted in a reduction in the number of eosinophils in bronchoalveolar lavage fluids, suppressed airway hyper-responsiveness after methacholine challenge, and resulted in lower levels of antigen-specific IgG1 and IgE (Suzukawa et al., 2012). Further highlighting its importance in promoting T_H_2 cell-mediated inflammation, IL-25 was found to promote the proliferation of human T_H_2 memory cells and enhanced their ability to produce T_H_2 cytokines after stimulation with TSLP-activated dendritic cells (DCs; Wang et al., 2007). Similarly, IL-33 has also been associated with asthma and the T_H_2 response. For example, higher levels of IL-33 transcripts were detected in biopsies from asthmatic compared with control subjects (Prefontaine et al., 2009). Further, adoptive transfer of dendritic cells pre-treated with IL-33 exacerbated lung inflammation in a DC-driven model of allergic airway inflammation demonstrating the importance of IL-33 in promoting asthma pathogenesis (Besnard et al., 2011). More recently, IL-25 and IL-33 promote the population expansion of IL-5- and IL-13-producing ILC2s that have been shown to promote pathogenesis in mouse models of allergic asthma and atopic dermatitis (Wolterink et al., 2012; Salimi et al., 2013). Therefore, similar to TSLP, IL-25, and IL-33 act upstream of T_H_2-associated cytokine and IgE production, making them enticing targets for biologics. However, although several murine studies have shown that targeting IL-25 and IL-33 with depleting antibodies may be effective in reducing airway inflammation and airway hyper-responsiveness, human trials have yet to yield promising results (Ballantyne et al., 2007; Liu et al., 2009). Nevertheless, the potential of anti-IL-25 and anti-IL-33 therapeutics to suppress multiple components of the T_H_2 response responsible for asthma pathogenesis warrants more attention.

## Summary

Asthma is an incredibly complex and heterogeneous disorder that affects millions of people around the world (Asthma, 2015a). Although the current pharmacological agents used to treat asthma, which include inhaled β2-adrenergic receptor agonists, inhaled corticosteroids, and anti-leukotrienes, are generally effective for the majority of asthma patients, there is a select group who are unresponsive (Chan et al., 1998; Chung and O'Byrne, 2003). In addition, most likely because of their unspecific mechanisms of actions, these therapies have been associated with serious side effects, especially if taken systemically or in large doses (Chung and O'Byrne, 2003; Dahl, 2006). For these reasons, it is imperative that new types of therapeutics to treat asthma are developed. Biologics may offer physicians a more personalized approach to treating allergy-associated asthma. In addition to the already FDA-approved biologics omalizumab and mepolizumab, therapies that target IL-13 are undergoing clinical trials (Soler et al., 2001; Lanier et al., 2003; Corren et al., 2011; Ingram and Kraft, 2012; Ortega et al., 2014). Critically, since recent studies have found that there are multiple classes of asthma, each with its own unique characteristics, it would be expected that certain biologics would be more effective than others in treating each phenotype (Wenzel, 2012). Perhaps the most exciting therapeutic target is the cytokine alarmin TSLP because of its ability to alter hematopoiesis and to promote inflammation via targeting both innate and adaptive immune cells (Soumelis et al., 2002; Ito et al., 2005; Siracusa et al., 2011; Kim et al., 2013). Additionally, because TSLP acts upstream of T_H_2-associated cytokine and IgE production, it may potentially act as a “master switch” in that inhibiting its actions may shut down multiple components of the T_H_2 response responsible for the initiation of asthma pathogenesis. Moreover, despite the lack of human studies, biologics that target IL-25 and IL-33 also have the potential suppress the T_H_2 response in a similar fashion and should not be neglected. Because biologics that target upstream components of the T_H_2 response are in the early stages of development, it is difficult to predict any complications that may arise. Since these molecules regulate multiple aspects of the T_H_2 response, their inhibition could lead to non-desired effects including increased susceptibility to certain types of infections, especially those caused by parasites. However, the potential of targeting these alarmins to treat asthma should not be understated. Although our understanding of the pathways that drive asthma is still incomplete, we are beginning to grasp the potential of using biologics to treat allergic airway inflammation. As additional pathways involved in asthma pathogenesis are uncovered, therapeutics that target these pathways are developed, which would aid in the eventual goal of creating personalized treatment regimens for each patient specific to his or her disease phenotype.

## Author contributions

CS and MS were responsible for the drafting and revising of the review article.

### Conflict of interest statement

The authors declare that the research was conducted in the absence of any commercial or financial relationships that could be construed as a potential conflict of interest.
